# Bochdalek hernia with retrocardiac spleen - Diagnostic dilemma for emergency care physicians—A case report

**DOI:** 10.1016/j.ijscr.2020.03.051

**Published:** 2020-05-14

**Authors:** Liaqat A. Khan, Ali M. Al-Neami, Ayman F. Soliman, Alaa A.M. Khaled, Mohammed I.H. Tawhari, Yahya A. Athlawy

**Affiliations:** a Department of Radiology, Baish General Hospital, Ministry of Health, Jazan, Saudi Arabia; b Department of General and Laparoscopic Surgery, Sabya General Hospital, Ministry of Health, Jazan, Saudi Arabia

**Keywords:** Bochdalek, Hernia, Retrocardiac, Spleen, Diaphragm, Physician, Emergency care

## Abstract

**Introduction:**

Physicians working as first-level responders in emergency departments (ED) often encounter patients, of any age group with shortness of breath (SOB). Definitive diagnosis is quite challenging once the underlying pathology is rare and unusual and/or the ED physicians recommend and rely on non-specific investigations.

**Presentation of case:**

A 29-year-old female presented to the emergency department with sudden onset of shortness of breath and upper abdominal pain radiating to the left shoulder. Diagnosis of Bochdalek hernia was made clinically coupled with radiological findings of CXR and computed tomography (CT) by the surgeon on-call, while it was missed by an emergency care physician on her first visit.

**Discussion:**

Herniation of the abdominal contents into the thoracic cavity via the Bochdalek opening, commonly known as Bochdalek hernia is seen and diagnosed most commonly accidentally in early life. Adult cases of symptomatic Bochdalek hernia has been reported in the literature. These patients usually present with non-specific symptoms, thus pose a diagnostic challenge for an emergency care physician.

**Conclusion:**

The report of this case highlights the notion that such rare causes of acute onset dyspnea and upper abdominal pain pose a diagnostic challenge for novice emergency care physicians especially in situations where he/she does not ask for second-line help in general and recommend and rely on a non-specific investigation in specific.

## Highlights

• Bochdalek Hernia is most commonly found in young age.
• Bochdalek Hernia may present in old age with non-specific symptoms.
• ER Physicians and surgeons should be critical in thinking keeping such unusual pathology in mind while dealing with patients.
• New ER Physicians commonly miss such rare and unusual cause of abdominal and thoracic symptoms.

## 1 Introduction

Emergency care physicians working as first-level responders encounter patients most often with sudden onset shortness of breath and upper abdominal pain, where it is mandatory to find the underlying pathology and plan case-specific management. Diagnosing the underlying pathology is most challenging in situations where the underlying pathology is rare and unusual, especially for ED physicians who rarely encounter such rare scenarios.

One of the extremely rare causes of shortness of breath and upper abdominal pain is sudden onset herniation of the abdominal contents into the thoracic cavity, a condition commonly known as Bochdalek hernia that was first reported by a Czech anatomist Vincent Alexander Bochdalek [[1], [2], [3]] in 1848. The condition results from failure of fusion at level 10th and 11th rib of the posterolateral diaphragmatic foramina commonly known as foramen of Bochdalek, an opening through which peritoneal and pleural cavities communicate [4]. The condition is commonly seen in the neonatal and early pediatric age groups and rarely in adult life.

Herein we report a young female patient with Bochdalek hernia associated with the retrocardiac spleen that was managed surgically. This work is reported in line with the SCARE criteria guidelines for case reports [5].

## 2 Case report

A 29 years old female, physician by profession presented to the emergency department with a history of aggressive vomiting five weeks back followed by left upper abdominal, a single episode of loose motion, subcostal pain radiating to left shoulder associated with shortness of breath (SOB) and was unable to take full inspiration. The patient has a history of heartburn, early satiety, indigestion, and food regurgitation six years ago and diagnosed and managed as gastroesophageal reflux disease in her native country.

**Fig. 1:**
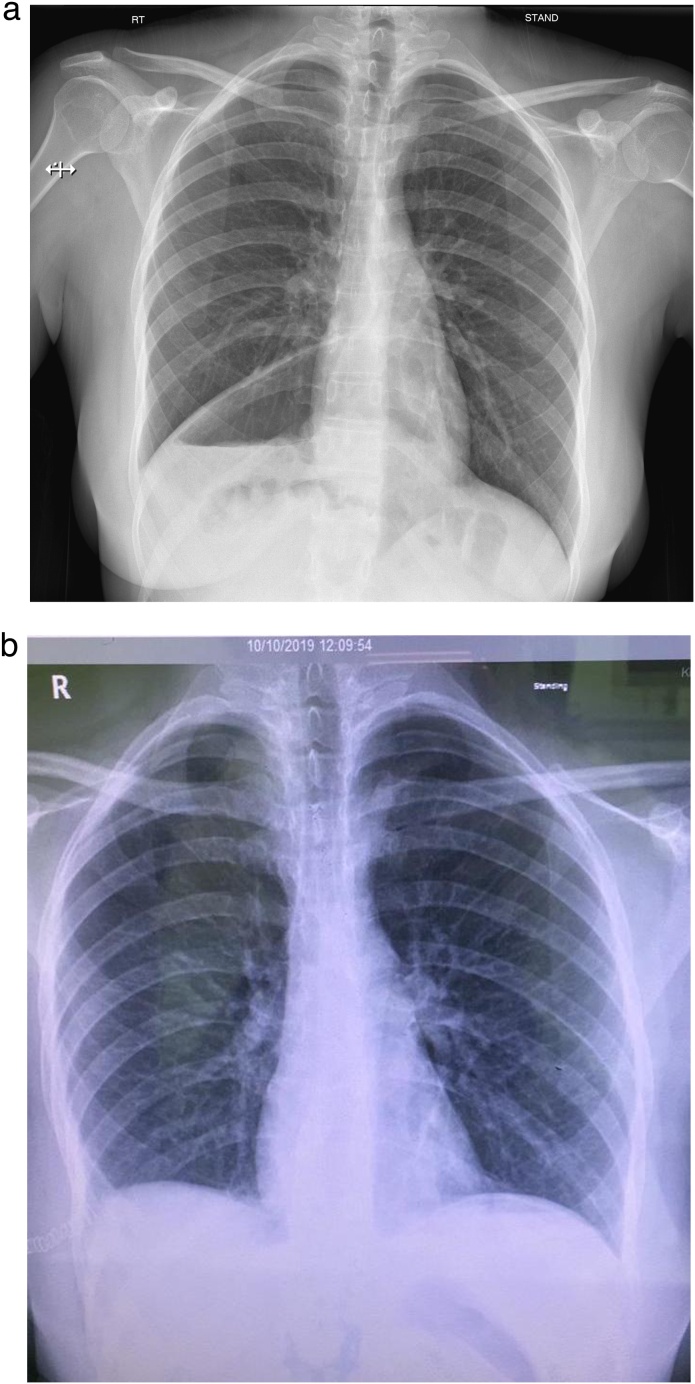
Pre-op x-ray chest with Chilaiditis Sign (a); Post-op x-ray chest (normal) (b).

The primary evaluation shows a toxic looking afebrile patient with vitals as; respiratory rate-27/min, pulse 87/min. Along with first-level management, abdominal ultrasonography (US) and initial laboratory workup were done in ED with no abnormal findings and the patient discharged home after the primary management independently and not asking surgeon on-call help. Upon no improvement, the patient revisited the ED where chest x-ray (CXR) as primary imaging modality was requested by surgeon on-call that showed raised right hemidiaphragm with no well discernible outlines, air-filled bowel loops above the hepatic shadow, a chilaiditi's sign, with no mediastinal shift (Fig. 1a), thus a provisional diagnosis of the right-sided diagrammatic hernia was made.

**Fig. 2:**
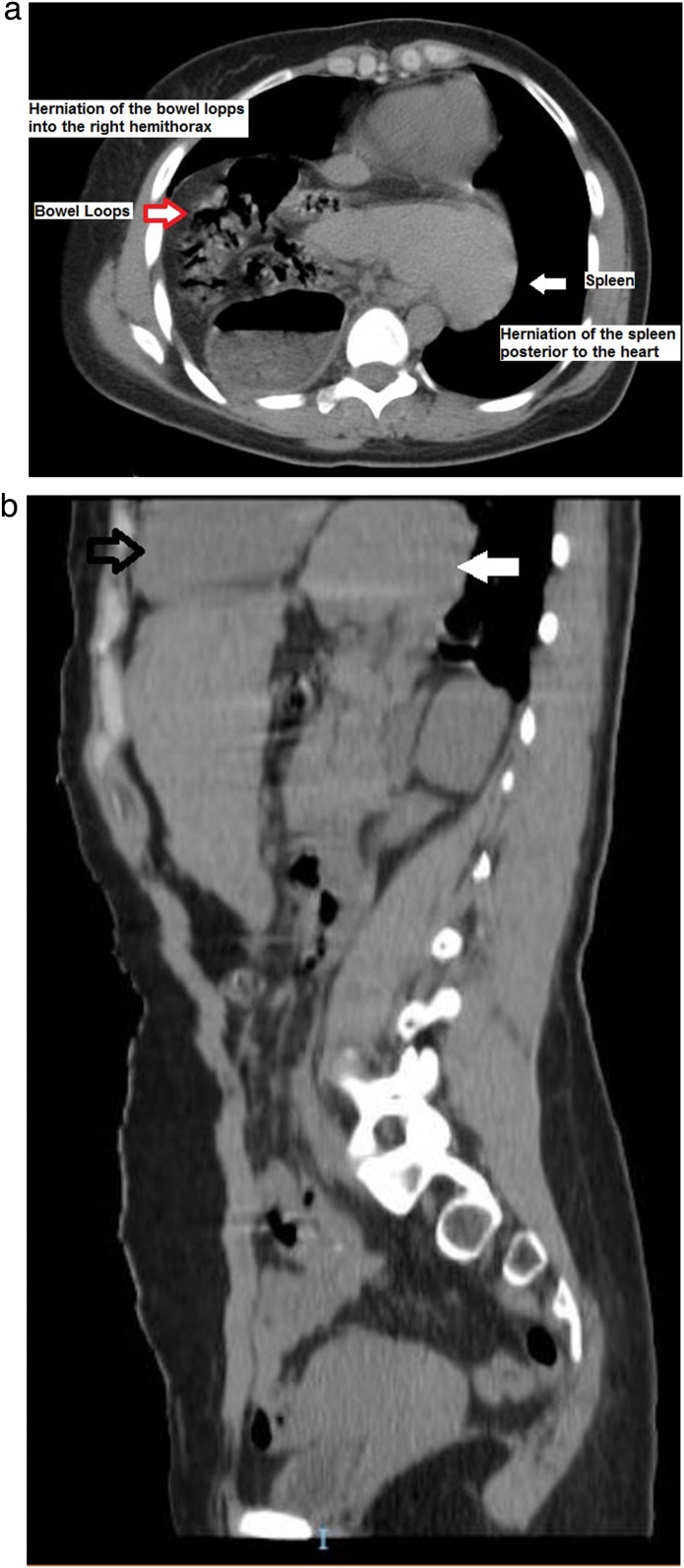
Non-contrast CT-Intrathorasic Extrusion of Spleen, Stomach and Bowels (a); Non-contrast CT-retrocardiac spleen (b).

Following CXR, a non-contrast CT requested showing stomach and parts of the colon in the right thoracic cavity (Fig. 2a) along with spleen located posterior to the heart – the retrocardiac spleen (Fig. 2b), thus a final diagnosis of Bochdalek hernia was made.

Severe vomiting, a few weeks earlier was the triggering event in the patient that led to the initiation of the clinical picture. After initial stabilization, the patient was transferred to a regional tertiary care facility for cardiothoracic surgeon evaluation and management where via open thoracotomy, contents reduced and fortunately there was no vascular compromise. Repair done. Post-operative chest x-ray shows normal findings (Fig. 1b). The patient had uneventful recovery and discharged home on 10th post-operative day. The patient did well in her follow-up period. To the best of our knowledge, it is the first reported case of Bochdalek hernia associated with the retrocardiac spleen in an adult female in the published literature.

## 3 Discussion

During embryonic life the pleuroperitoneal folds fail to close, resulting in an opening called Bochdalek opening through which the abdominal contents herniate into the thoracic cavity commonly known as Bochdalek hernia. The condition is most commonly manifest in early weeks of life while adult cases of symptomatic Bochdalek hernia (ABH) have also been reported in the published literature. The four types of congenital diaphragmatic hernia include parasternal hernia of Morgagni-Larrey, peritoneo-pericardial hernia, eventration of the diaphragm and the posterolateral hernia of Bochdalek [6] commonest in all. In 85% of the cases, the hernia is on the left side through which the spleen, stomach, the small and large intestine may herniate as seen in this case with stomach and spleen herniation in the thoracic cavity and less commonly right-sided hernia do occur [7,10].

Many patients remain asymptomatic until adult life or may present with chronic symptoms of abdominal or respiratory such as recurrent abdominal or chest pain, early satiety, and fullness after meal or vomiting [4,8]. Potential complications include vascular compromise of hernial contents, pneumo or haemothorax [9], and fortunately, the patient has no such complication. Diagnosis of ABH is quite challenging and a 38% failure rate has been reported by Thomas et al. [10], as in this case the diagnosis was missed by the ED physician on the patient's first visit. Computed tomography scan (CT) with contrast media has sensitivities as high as 78% in left-sided and 50% in right-sided ABH and can diagnose herniation more accurately as compared to plain x-ray chest [11]. Management of ABH includes surgical reduction of abdominal contents via, laparotomy, thoracotomy or laparoscopic repair, each approach has its pros and cons while the latter is preferred due to short hospital stay and low complication rate [12].

## 4 Conclusion

Rare and unusual causes of acute onset dyspnea in adults with or without abdominal pain pose a diagnostic change for novice emergency care physicians. A high index of suspicion, clinical expertise and second-line help coupled with a basic radiological investigation like CXR and advanced radiological services such as CT scan are useful in dealing with patients with dyspnea and upper abdominal pain to reach a definitive diagnosis and plan case-specific management.

## Declaration of Competing Interest

The Authors declare that they have no Conflict of Interest.

## Funding

Nil to declare.

## Ethical approval

As it is a case study, thus no prior ethical approval obtained.

## Consent

Written Informed Consent taken from patient for publication of this case with accompanying images.

## Author contribution

Liaqat A. Khan: Conceptualization, data interpretation, writing, reviewing and editing of the manuscript.

Ali M. Al-Neami: Conceptualization, reviewing and editing the draft.

Ayman F. Soliman: Acquisition of data, reviewing the manuscript.

Alaa A.M. Khaled: Roles/writing, original draft.

Mohammed I.H. Tawhari: Editing the original draft.

Yahya A. Athlawy: Reviewing the final draft.

All authors approved the final version of the manuscript.

## Registration of research studies

As this is a case report and not an interventional study, this manuscript is exempt from registration.

## Guarantor

Ali M. Al-Neami.

## Provenance and peer review

Not commissioned, externally peer-reviewed.
